# Toward robust and scalable deep spiking reinforcement learning

**DOI:** 10.3389/fnbot.2022.1075647

**Published:** 2023-01-20

**Authors:** Mahmoud Akl, Deniz Ergene, Florian Walter, Alois Knoll

**Affiliations:** Chair of Robotics, Artificial Intelligence and Embedded Systems, TUM School of Computation, Information and Technology, Technische Universität München, Munich, Germany

**Keywords:** spiking neural network (SNN), reinforcement learning, deep reinforcement learning (Deep RL), continuous control, hyperparameter tuning

## Abstract

Deep reinforcement learning (DRL) combines reinforcement learning algorithms with deep neural networks (DNNs). Spiking neural networks (SNNs) have been shown to be a biologically plausible and energy efficient alternative to DNNs. Since the introduction of surrogate gradient approaches that allowed to overcome the discontinuity in the spike function, SNNs can now be trained with the backpropagation through time (BPTT) algorithm. While largely explored on supervised learning problems, little work has been done on investigating the use of SNNs as function approximators in DRL. Here we show how SNNs can be applied to different DRL algorithms like Deep Q-Network (DQN) and Twin-Delayed Deep Deteministic Policy Gradient (TD3) for discrete and continuous action space environments, respectively. We found that SNNs are sensitive to the additional hyperparameters introduced by spiking neuron models like current and voltage decay factors, firing thresholds, and that extensive hyperparameter tuning is inevitable. However, we show that increasing the simulation time of SNNs, as well as applying a two-neuron encoding to the input observations helps reduce the sensitivity to the membrane parameters. Furthermore, we show that randomizing the membrane parameters, instead of selecting uniform values for all neurons, has stabilizing effects on the training. We conclude that SNNs can be utilized for learning complex continuous control problems with state-of-the-art DRL algorithms. While the training complexity increases, the resulting SNNs can be directly executed on neuromorphic processors and potentially benefit from their high energy efficiency.

## 1. Introduction

Spiking Neural Networks (SNNs), also known as the third generation of neural networks (Maass, 1997; Walter et al., 2016), have been studied as alternative universal function approximators to Artificial Neural Networks (ANNs). The biological plausibility of SNNs, as well as the orders of magnitude increased energy efficiency, especially when deployed on neuromorphic chips (Roy et al., 2019), are two main factors that contribute to the increasing interest in SNNs. Other factors, like the ability to process high-dimensional data in real time, particularly when data is provided from asynchronous sensors like event-based cameras (Gallego et al., 2022), provide SNNs with an edge over ANNs in particular applications.

For a long time, training SNNs has been limited to biologically plausible learning rules (Gerstner et al., 1993; Ruf and Schmitt, 1997), like spike time-dependent plasticity (STDP), or to evolving synaptic weights using genetic algorithms (Floreano and Mattiussi, 2001; Batllori et al., 2011; Schuman et al., 2020). While the backpropagation algorithm is not biologically plausible (Crick, 1989; Marblestone et al., 2016; Whittington and Bogacz, 2019), it has been proven to be a powerful tool when it comes to optimizing parameters in ANNs (LeCun et al., 2015). The main hurdle against training SNNs with backpropagation is the discontinuity in the spike function that renders it not differentiable. The gradient is infinite at the spiking threshold and zero everywhere else. For this reason, a lot of research focused on converting trained ANNs to SNNs, instead of directly training SNNs, to leverage the low power consumption of neuromorphic chips. This has been explored on supervised (Rueckauer et al., 2017; Sengupta et al., 2019; Han et al., 2020; Stöckl and Maass, 2021) as well as on reinforcement learning problems (Patel et al., 2019).

In the past few years, however, multiple techniques to approximate gradients in SNNs have been suggested. Here, we consider surrogate gradients (Bohte et al., 2002; Neftci et al., 2019), a method that replaces the discontinuous gradient function of the heaviside spike function with a smoothed one. Multiple surrogate gradient functions have been explored, e.g., Piece-wise Linear (Esser et al., 2016), derivative of a fast sigmoid (SuperSpike) (Zenke and Ganguli, 2018), and exponential (Shrestha and Orchard, 2018). However, it was found that surrogate gradient functions that peak at zero and are monotonically falling on both sides are similarly effective (Zenke and Vogels, 2021). Surrogate gradient learning has been heavily explored on classification problems (Bellec et al., 2018), but much less on reinforcement learning problems.

In a previous work (Akl et al., 2021), we demonstrated that SNN training with backpropagation and surrogate gradients can be combined with the Deep Q-Network (DQN) algorithm (Mnih et al., 2015) to solve classical control tasks from OpenAI Gym (Brockman et al., 2016). Furthermore, we showed that taking certain constraints into account during training allows us to port the trained networks to Intel's neuromorphic research chip Loihi (Davies et al., 2018) without loss in performance. In a follow-up work (Akl et al., 2022), we were able to fine-tune the trained SNNs with backpropagation and surrogate gradients using the biologically plausible reward-modulated STDP (r-STDP) learning rule, to restore the network's performance when evaluated on randomized versions of the environments.

In this paper, we further investigate using SNNs as function approximators in DRL algorithms. We expand on our previous work by training more advanced DRL algorithms with SNNs to solve more complex, continuous control problems from OpenAI Gym (Brockman et al., 2016), with an increased number of state and action dimensions. Additionally, we conduct a hyperparameter study in order to examine how the choice of the membrane parameters affects learning with surrogate gradients and DRL algorithms. Furthermore, we explore randomizing membrane parameters across the entire network, and observe that this approach improves SNN training with surrogate gradients.

The rest of this paper is organized as follows. In the next section, we describe the methods used to train SNNs, i.e., the neuronal model and the encoding and decoding methods as well as the randomization of the membrane parameters. In Section 3, we elaborate on the choice of membrane parameters and show how they affect trainability of the networks by conducting hyperparameter searches. In Section 4, we present the training results and highlight the impact of randomizing parameters. Finally, we conclude our work and determine potential future research directions in Section 5.

## 2. Methods

In this section we describe the methods we used to train SNNs. In particular, we elaborate on the neuronal model used, the surrogate gradient function, the DRL algorithm used to train the networks, our chosen encoding and decoding methods, as well as our approach to randomize membrane parameters.

### 2.1. SNN training with surrogate gradients and TD3

To train the SNNs with backpropagation and surrogate gradients, we used the SpyTorch framework (Zenke, 2019) which is built on top of the popular deep learning library PyTorch (Paszke et al., 2019). We used the leaky integrate-and-fire (LIF) neuron model in all our experiments. In SpyTorch, the LIF model membrane dynamics in feed forward networks are described by:


(1) $$\begin{array}{l} V_{i}\left(t\right)=\beta V_{i}\left(t-1\right)+I_{i}\left(t-1\right)-S_{i}\left(t-1\right) \end{array}$$

where *V*_*i*_(*t*) is the membrane potential of neuron *i* at time *t*, $\beta =e^{-1/\tau_{mem}}\in \left[0,1\right]$ is the membrane potential decay factor that depends on the membrane time constant τ_*mem*_>0. *I*_*i*_(*t*) is the input synaptic current of neuron *i* at time *t*. *S*_*i*_(*t*) represents the emission of a spike once the membrane potential exceeds the firing threshold θ, and is formally described by the Heaviside step function:


(2) $$\begin{array}{l} S_{i}\left(t\right)=\Theta \left(U_{i}\left(t\right)-\theta \right) \end{array}$$

The input synaptic current is described by:


(3) $$\begin{array}{l} I_{i}\left(t\right)=\alpha I_{i}\left(t-1\right)+\underset{j}{\sum }W_{ij}S_{j}\left(t-1\right) \end{array}$$

Where $\alpha =e^{-1/\tau_{syn}}\in \left[0,1\right]$ is the synaptic current decay factor that depends on the synaptic time constant τ_*syn*_>0, and $\underset{j}{\sum }W_{ij}S_{j}\left(t-1\right)$ is the weighted sum of the incoming spikes from the previous layer *j*.

The DRL algorithm we considered for the continuous control problems is TD3 (Fujimoto et al., 2018). It is an extension of the off-policy Deep Deterministic Policy Gradient (DDPG) (Lillicrap et al., 2016) algorithm. With TD3, three new methods were introduced in order to deal with the often-encountered *Q*-value overestimation problem: Target policy smoothing, double Q-learning, and delayed policy updates. TD3 is an actor critic method in which an actor network is trained to output actions based on current observations, and two critic networks are trained to estimate the action value, based on the current observation. All networks used in our experiments are feed-forward networks with two hidden layers containing 400 and 300 neurons, respectively. We chose the actor network to be an SNN and the critic networks to be ANNs. While using SNNs for the actor and critic networks is feasible, there is a notable boost in training times when choosing ANN critics. This is mainly due to the fact that current deep learning frameworks that are used to train SNNs with surrogate gradients are not optimized for SNNs or sparse computations. Finally, once an agent is trained, only the actor network is used for evaluation and deployment. If our end goal is to deploy a trained DRL policy to neuromorphic chips in order to leverage low power consumption, then only the actor network needs to be spiking. Furthermore, training a TD3 agent with spiking critic networks might require a different set of membrane parameters than that of the actor network. This will require further hyperparameter tuning of the critics' membrane parameters.

### 2.2. Encoding and decoding

In continuous control RL problems, observations are real-valued sensor readings and actions are real-valued torques applied to joints. In order solve such problems with SNNs, suitable encoding and decoding methods have to be chosen. While encoding and decoding information is a heavily studied problem in the field of SNN research (Schuman et al., 2019, 2022; Auge et al., 2021; Guo et al., 2021), knowing which encoding and decoding methods (or the combination thereof) are suitable for particular applications is seldom straightforward.

**Figure 1:**
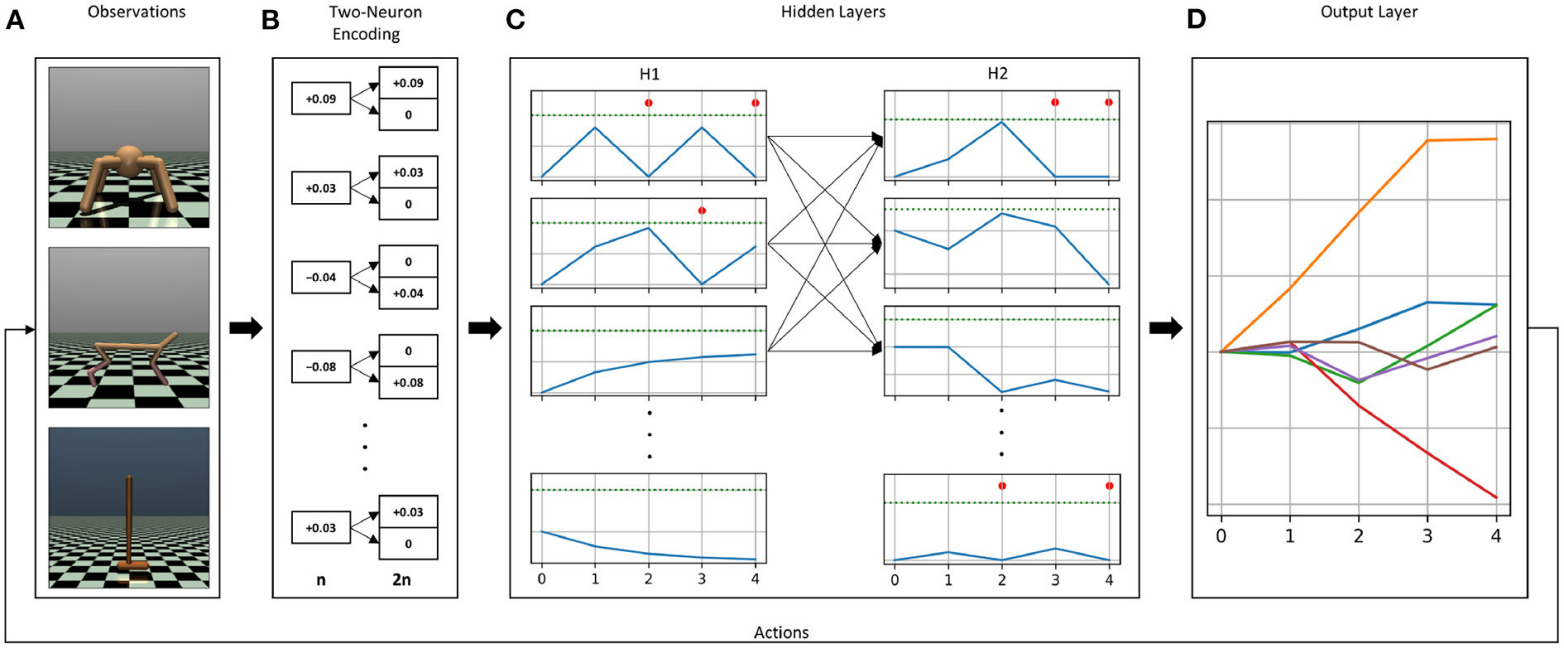
Overview of the encoding and decoding methods used in our experiments. The continuous control OpenAI Gym environments Ant-v3, HalfCheetah-v3, and Hopper-v3 **(A)**. Each environment has different observation and action dimensions, which has an impact on the choice of the membrane parameters. Observations are first encoded into a two-neuron input scheme, in which inputs are split into positive and negative neurons for each dimension **(B)**. The resluting two-neuron encoded input vector is multiplied by the first weight matrix, i.e., the weight matrix connecting the input layer to the first hidden layer, and the resulting values are injected as constant current in the first hidden layer's neurons. **(C)** Shows the resulting activity from injecting constant current. Blue curves show the membrane potential, dashed green lines represent the firing thresholds, and the red dots indicate the emission of a spike at that time step. Spikes generated in the first hidden layer will propagate to the second hidden layer, where spiking activity will also be generated. The output layer **(D)**, contains as many neurons as the number of actions with the firing threshold set to infinity, i.e., neurons without a spiking mechanism. Incoming spikes from the second hidden layer control the evolution of the membrane potentials of the neurons in the output layer. The values of the membrane potentials at the last simulation time step are the chosen actions.

Previously, for discrete action problems, we used the current injection of weighted sum of inputs encoding method alongside the membrane potential decoding method. The combination of both methods was able to solve classic control tasks when trained with the DQN algorithm. With slight modifications, we were able to re-use the same methods for continuous control problems. A schematic overview of our chosen encoding and decoding methods for continuous control is shown in Figure 1.

#### 2.2.1. Current injection of observations' weighted sum

In this input encoding method, the observations from the environments are multiplied by the first weight matrix (connecting the input layer to the first hidden layer). The values in the resulting vector are injected as constant current in the first hidden layer's neurons for the entire duration of the simulation time. With this approach, we are using a linear ANN input layer, and allow the trained weights to adjust what amount of current gets injected into the spiking neurons. One of the main advantages of this approach, as opposed to injecting observations as currents in the input layer directly, is that we never have to worry about whether the observations' values are high enough to generate spiking activity or not. It is standard practice in DRL to normalize observations before feeding them into the network, and injecting normalized observations as current can lead to a dead network, especially at the beginning of an episode, where input values like velocities and joint positions are low. In continuous control problems, observations are real values spanning negative and positive numbers, which usually requires a transformation before translating them into spike trains. The main advantage of this encoding method is that it does not require any transformation to the input before feeding it into the network. Since weights are initialized from a normal distribution centered around zero, the weight matrices contain positive as well as negative values. Therefore, negative observations multiplied by negative weights will result in positive constant currents, which, if high enough, will produce spiking activity. Similarly, some observations will result in negative current injection in some neurons, which will cause the membrane potential to fall below its resting value, i.e., sub-resting membrane potential (see for example the last neuron in the first hidden layer H1 in Figure 1C). While this feature of sub-resting membrane potential is sometimes ignored in software and hardware implementations of SNNs, it is a biologically realistic feature that has been shown to result in more accurate inference in SNNs (Hwang et al., 2020).

Even though a transformation of the inputs is not required in our case, we found that applying a two-neuron encoding on the raw environment observations (See Figures 1A, B), has stabilizing effects on the training. The two-neuron encoding method assigns two neurons, instead of one, for each observation. One neuron gets activated when the observation is positive, while the other neuron gets activated when the observation is negative. The two neurons representing one observation dimension produce mutually exclusive firing, i.e., only one of them gets activated at a time (see Figure 1B). This method was first introduced in Pérez-Carrasco et al. (2013) in order to be able to convert negative inputs to spike trains.

#### 2.2.2. Membrane potential readout

In this decoding method, we remove the spiking mechanism from the output neurons, i.e., we set the firing threshold of the output neurons to infinity. This way, the output neurons never spike, regardless how high the membrane potential gets. Incoming weighted spikes increase or decrease the values of the membrane potentials. The final values of the membrane potentials of the output neurons, i.e., after the last simulation time step are chosen as actions (See Figure 1D). Unlike discrete action problems, in which only a higher value is sufficient to select an action, continuous control problems require accurate readout values that translate into meaningful actions. We found that setting the current decay factor to zero α = 0 and the voltage decay factor to one β = 1 in the output layer yielded more stable training results for continuous control problems. By doing so, our output neurons are calculating the weighted sum of incoming spikes, without any leak.

### 2.3. Randomized membrane parameters

One assumption that is often considered when building neural networks, is that all neurons share the same parameters. In ANNs, this means that all neurons have the same activation function, while in SNNs this means that all neurons have the same membrane parameters. Broadly speaking, this assumption means that all neurons within a network would produce the same output when subjected to the same input. However, neurons in different brain regions have been shown to have different time constants (Deco et al., 2019), which would cause diverse output activities. In an attempt to incorporate more neuroscience findings into (artificial) neural networks, we explored the trainability of SNNs with non-uniform membrane parameters, when trained with backpropagation based on surrogate gradients and the TD3 DRL algorithm to solve continuous control porblems.

Previous works considered treating membrane time constants as learnable parameters that are optimized during training alongside synaptic weights (Zimmer et al., 2019; Fang et al., 2021). There, all neurons within a layer share the same time constants. This approach showed an improved classification accuracy of SNNs when measured on several benchmark datasets. Another work (Perez-Nieves et al., 2021) compared the effects of initialized uniform parameters that are modified during learning, with initialized random parameters that are fixed during training. Overall, introducing randomized membrane parameters was shown to improve the accuracy to various degrees. The authors argue, that for tasks with rich intrinsic temporal structure, heterogeneity was most effective. In this paper, however, we consider randomizing the membrane parameters' values for each neuron during initialization, and keep the values fixed, while only optimizing the synaptic weights to solve continuous control problems. Additionally, we randomize the firing thresholds as well as the time constants (current and voltage decay factors). When initializing a network, we draw the respective values for each neuron from a narrow normal distribution, centered around the chosen values for each parameter, i.e., the values listed in **Table 2**, with the standard deviation set to σ = 0.1·μ.

**Figure 2:**
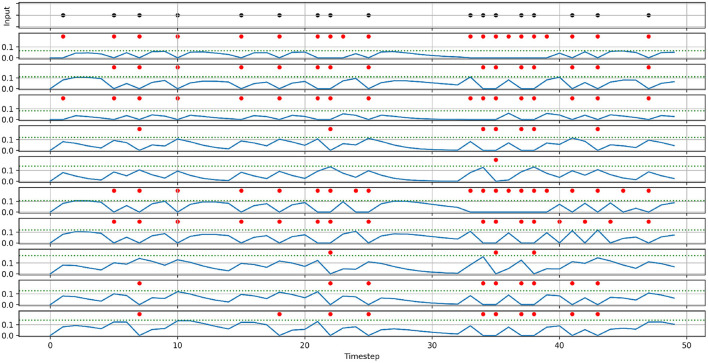
Demonstration of the effects of randomizing membrane parameters. An input spike train **(top)** is fed into ten neurons with the same synaptic weight. Instead of setting the membrane parameters' values of all neurons to the same value, the values are drawn from a normal distribution with the means: μ_α_ = 0.5, μ_β_ = 0.5, μ_*threshold*_ = 0.1, and the standard deviation: σ = 0.3μ. The panels below the input spike train show the membrane potentials (solid blue lines), thresholds (dashed blue lines), and the spikes (red dots) of the ten neurons.

To demonstrate the effect of randomizing membrane parameters, Figure 2 shows the activity of 10 neurons with randomized parameters, that receive the same spike train input and share the same synaptic weight. For demonstration purposes, we set the standard deviation to a higher value than in our experiments (0.3·μ vs. 0.1·μ). The most notable effect of randomizing membrane parameters is that every neuron produces a different number of spikes, and thereby has a different firing rate. Additionally, looking at the membrane potentials, we can also see how the neurons have different decay behaviors (most notably around timestep 30), which in turn also affects the firing rate.

## 3. Impact of membrane parameters

One of the reasons why training SNNs is more difficult than training ANNs is that SNNs have an increased number of parameters, the choice of which heavily impacts the learning ability. In ANNs, apart from the DRL algorithm hyperparameters (e.g., memory buffer size, target update frequency, discount factor, added noise to selected actions, and epsilon greedy action selection), and the gradient descent hyperparameters (e.g., learning rate and batch size), the neuron-specific parameters are limited to the choice of the activation function. The activation function is a mapping that defines the output produced by a neuron based on its input. In SNNs, the equivalent of an activation function is the set of membrane parameters that define the neuron's dynamics and describe how the neuron's current and voltage evolve in response to stimuli. In a leaky integrate-and-fire (LIF) neuron model (Lapique, 1907; Gerstner et al., 2014), the membrane parameters include the voltage decay factor, the syanptic current decay factor, and the firing threshold. While other parameters may also be adjusted, they are often set to default values. These parameters can also impact the trainability of the network using backpropagation and surrogate gradients. For example, the value of the reset membrane potential, i.e., the value the membrane potential takes after a neuron emits a spike, and the refractory period, i.e., the period of time where a neuron cannot spike after having emitted a spike, are set to zero by default in the SpyTorch LIF model, and we did not change them. In addition, other neuron models may have additional parameters that also need to be adjusted. For instance, LIF neurons with adaptive threshold (Chacron et al., 2003) have additional parameters such as the value by which the threshold increases after a spike is emitted and the threshold's decay factor. Tuning these parameters can affect the performance of the model.

In order to better understand how the choice of the membrane parameters impacts the backpropagation-based learning in SNNs, and specifically when combined with DRL algorithms, we ran extensive hyperparameter searches on taining an SNN with the DQN algorithm to solve the CartPole-v0 problem from OpenAI Gym (Brockman et al., 2016). We chose CartPole-v0 for this hyperparameter study since we had to train a lot of models to explore the parameter space, and CartPole-v0 is known of it's low complexity when compared to other OpenAI Gym environments. We performed a grid search over the membrane parameters of the LIF neuron model (voltage decay factor, current decay factor, firing threshold, and simulation time) when training an SNN with the DQN algorithm to solve the CartPole-v0 problem, while keeping the network's architecture and the DQN parameters fixed, as well as the random seeds controlling the weight and environment initialization. In SpyTorch, initial network weights are drawn from a gaussian distribution with a zero mean and a standard deviation defined according to:


(4) $$\begin{array}{l} \sigma_{ij}=\frac{weight\text{\_}scale}{\sqrt{N_{i}}} \end{array}$$

Where σ_*ij*_ is the standard deviation for the weight matrix connecting layer *i* to layer *j*, *N*_*i*_ is the number of neurons within layer *i*, and *weight*_*scale* is an additional parameter. While *weight*_*scale* can be chosen to depend on the decay factor values, we set *weight*_*scale* = 1 in all our experiments, in order to explore the effects of the membrane parameters on the trainability on the networks in isolation.

For each set of membrane parameters we trained the DQN for 1,000 episodes and saved the maximum reward that was achieved. The reward is measured as the average reward achieved over 100 consecutive episodes. However, the large number of possible parameter combinations made it necessary to set some constraints to make the search feasible. To do this, we set the voltage and current decay factors to the same value: α = βϵ[0 − 1]. In our previous experiments (Akl et al., 2021), we used the same values for α and β when solving the CartPole-v0 and the Acrobot-v1 problems, and found that this choice yielded good training results. In the discretized SpyTorch formulation of SNNs, α and β can take values in the range [0, 1]. We kept the same range for the threshold, as we found that values larger than one yield worse results. However, this choice is specific to the CartPole-v0 problem with its four observations, and other problems with more observations may require a higher threshold value, as more observations generate more activity. Furthermore, the defined ranges for each parameter are specific to the encoding and decoding methods chosen here. Different methods may require different values in order to generate spiking activity in the network and produce meaningful readout values.

**Figure 3:**
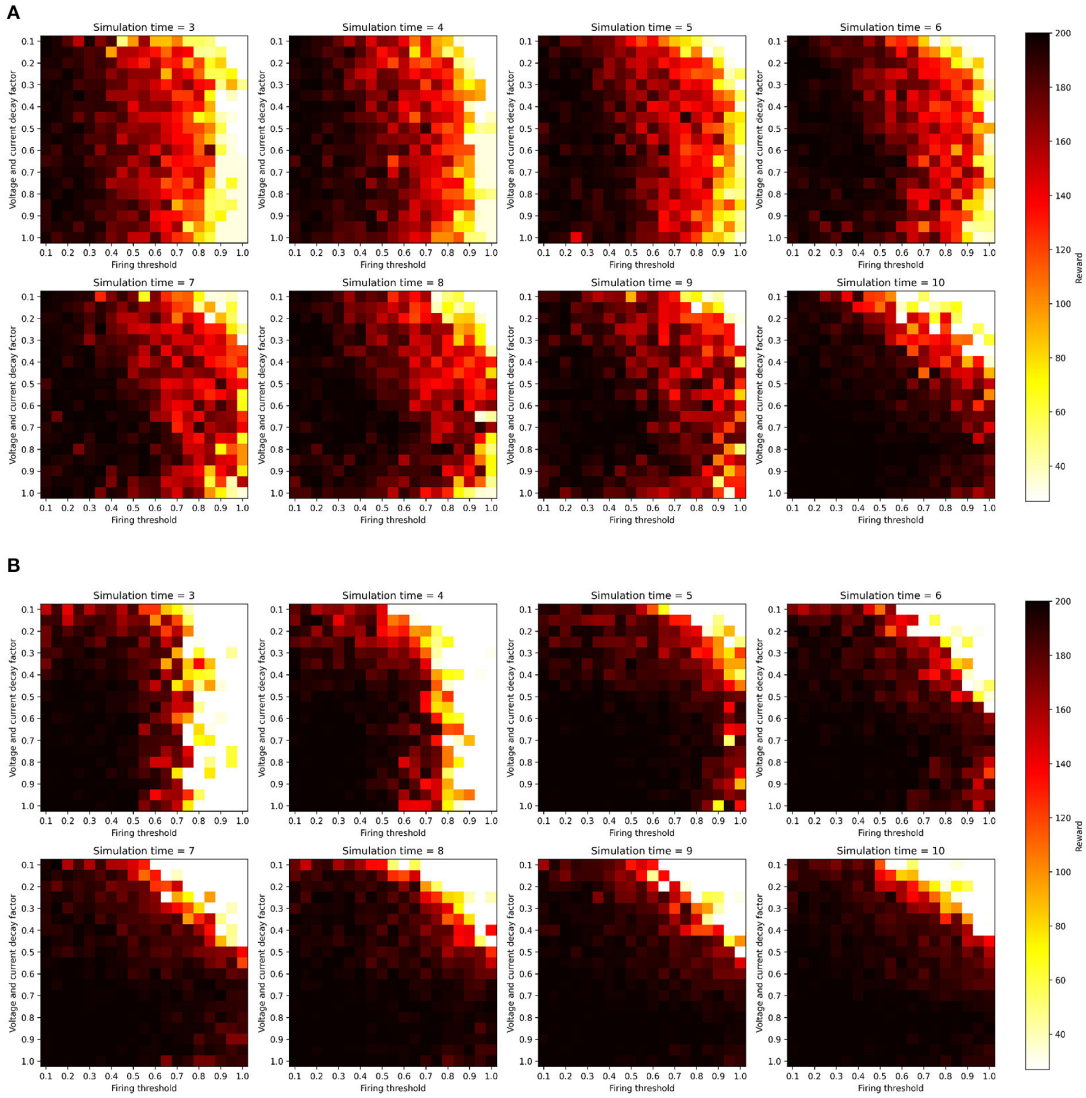
SNN hyperparameter search results over firing threshold and decay factor combinations for multiple simulation times, measured on the CartPole-v0 problem when trained with the DQN algorithm. All parameter combinations were used to train SNNs for 1,000 episodes, and the z-axis shows the maximum reward achieved during training. All experiments were conducted with the same random seeds controlling network weight initialization and environment's initial configuration. **(A)** One-neuron encoding. **(B)** Two-neuron encoding.

**Figure 4:**
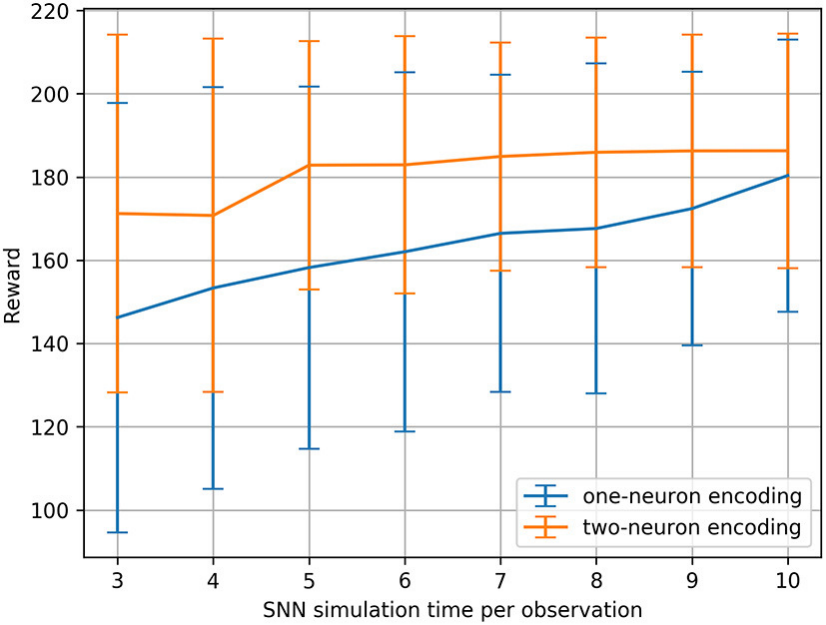
CartPole-v0 reward means and standard deviations measured across all membrane parameter combinations (for the defined ranges) for different SNN simulation times using one-neuron and two-neuron encoding. Running a statistical *t*-test on the rewards resulted in the significance values: *t* = −8.17, *p* < 0.001.

We ran this hyperparameter grid search twice, once with the current injection encoding method described in Section 2.2.1, and once with an additional two-neuron encoding to investigate the effects of applying such an input transformation to the sensitivity of the SNNs. Figure 3 shows the results of running the membrane parameter search on the CartPole-v0 problem for multiple simulation times. The minimum simulation time we can use that would allow spikes to propagate to the output layer is three (based on the number of layers and our input encoding method). The upper limit for the simulation time we chose was ten, as we observed that the network becomes immune toward the chosen membrane parameters and can achieve the maximum reward across most parameter combinations. The only parameter combination that limits the network's trainability even for large simulation times (e.g., 10) is low decay factor values and large threshold values. This combination does not produce enough spiking activity within the network, and thus the membrane potentials of the output neurons (i.e., the *Q*-values), remain at zero. One important observation is that increasing the simulation time helps reduce the network's sensitivity to the hyperparameters. Figure 4 shows the average reward across all parameter combinations for different simulation times. It is clear, that allowing the network more timesteps to process an observation yields improved robustness to the chosen membrane parameters. However, increasing the simulation time comes at the cost of efficiency, especially when training SNNs on CPUs and GPUs using deep learning frameworks that are not optimized for sparse computations, rather than on neuromorphic processors. While training SNNs with backpropagation has been explored on the Loihi neuromorphic chip. Backpropagation Algorithm Implemented on Spiking Neuromorphic Hardware (Renner et al., 2021). It was only demonstrated for a shallow network and the approach does not scale up to more complex network architectures.

An additional analysis we conducted on the results of the hyperparameter grid search, is to measure the percentage of parameter combinations that were able to successfully solve the problem for different simulation times. CartPole-v0 has a reward threshold of 195, meaning that agents achieving a reward greater or equal to 195 are considered to have solved the problem. Table 1 lists the percentages for the one-neuron as well as the two-neuron encoding methods across the different simulation times considered. Those percentages confirm the previous result, that increasing the simulation time in the one-neuron encoding case reduces the sensitivity to the hyperparameters. However, we only witness a large increase in the percentage when setting the simulation time to 10. Similarly, in the two-neuron encoding case, we also see that increasing the simulation time reduces the sensitivity. Nevertheless, we can also see that applying the two-neuron encoding can help us reduce the simulation time, while still expecting the same results. For example, the percentage of parameter combinations in the two-neuron encoding case that are able to solve CartPole-v0 with a simulation time of five exceeds that of the one-neuron case with a simulation time of 10 (the highest percentage among the one-neuron encoding). Those findings motivated the choice of our parameters for the continuous control problems discussed in the next section.

**Table 1:** Reward threshold crossing percentages across all parameter combinations for different simulation times.

**Simulation time**	**One-neuron encoding**	**Two-neuron encoding**
3	12.46%	30.74%
4	15.78%	31.02%
5	20.22%	48.19%
6	24.93%	43.49%
7	26.59%	42.10%
8	26.86%	49.58%
9	27.70%	**52.07%**
10	**43.49%**	48.47%

Values in bold indicate the highest percentage of reward threshold crossings.

## 4. Training results

We trained SNNs with backpropagation based on surrogate gradients and the TD3 algorithm (Fujimoto et al., 2018) as described in Section 2.1 to solve the Ant-v3, HalfCheetah-v3, Hopper-v3, and the Pendulum-v0 environments from OpenAI Gym. Those are popular continuous-action benchmark environments in DRL research and have been considered in various works before (Haarnoja et al., 2018; Kumar et al., 2019; Agarwal et al., 2020). The observation space, the action space, as well as the reward definition varies across all environments, and the exact details of all environments can be found in the official OpenAI Gym documentation^1^.

On each environment we trained spiking agents with uniform membrane parameters and randomized membrane parameters. Since the chosen environments have varying state and action space dimensions, we had to select different sets of membrane parameters for each environment. The uniform membrane parameters for all environments are listed in Table 2. In the randomized parameters case, we used the values listed in Table 2 as means, and drew the neuron-specific values from a normal distribution with a standard deviation of σ = 0.1·μ. Based on the findings of our hyperparameter study, we applied a two-neuron encoding for all environments, and set the simulation time to five steps. Each observation is first normalized, then transformed into a two-neuron representation and is fed into the network through constant current injection for the entire duration of the simulation time (five time steps). At the last time step, the membrane potential values of the output neurons are chosen as the actions for the next environment step. After simulating the SNN for one observation, the network state is reset by setting all neurons' membrane potentials, synaptic currents and spikes to zero.

**Table 2:** SNN parameters (current decay, voltage decay, firing threshold, and simulation time) used during training for all environments.

**Parameter**	**Ant-v3**	**HalfCheetah-v3**	**Hopper-v3**	**Pendulum-v0**
α	0.5	0.3	0.5	0.5
β	0.5	0.3	0.5	0.5
Threshold	2.5	0.8	2.0	1.0
Simulation time	5	5	5	5

**Figure 5:**
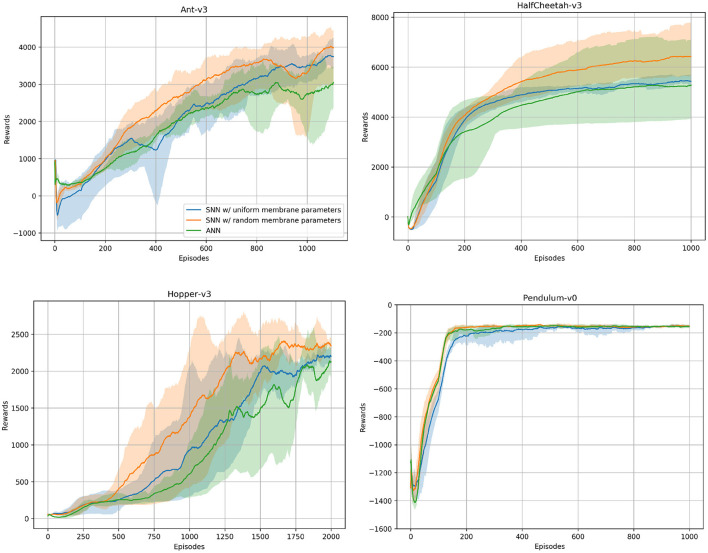
ANN and SNN training curves on the Ant-v3, HalfCheetah-v3, Hopper-v3, and Pendulum-v0 environments from OpenAI Gym. Two SNN training curves are to be seen, one using uniform and the other one using randomized membrane parameters. Solid lines are mean rewards (window size of 100 episodes) averaged over five runs with random initialization seeds. Shaded areas show the standard deviation. The number of episodes varies across environments, as each environment might have a different maximum number of timesteps per episode, i.e., horizon.

In addition to the spiking agents, we trained ANN agents with TD3 and the same hyperparameters to compare the SNN results to. We used the same network architecture for all environments. Our networks consist of two hidden layers containing 400 and 300 neurons, and we trained the networks on all environments for one million timesteps. For each environment we trained five models using different random seeds. Figure 5 shows the ANN and SNN training results on the Ant-v3, HalfCheetah-v3, Hopper-v3, and Pendulum-v0 environments using uniform and randomized membrane parameters. In all environments, the ANN training results are competitive with the uniform SNN training results. Using randomized membrane parameters, however, yielded higher averaged mean rewards. We measured the significance values of the rewards achieved during training with uniform and random membrane parameters in SNNs based on the dependent *t*-test for paired samples, and the results are summarized in Table 3.

**Table 3:** Random membrane parameters significance values.

**Environment**	**Significance**
Ant-v3	*t* = −36.39, *p* < 0.001
HalfCheetah-v3	*t* = −53.95, *p* < 0.001
Hopper-v3	*t* = −50.62, *p* < 0.001
Pendulum-v0	*t* = −39.63, *p* < 0.001

*P*-values are calculated based on the dependent *t*-test for paired samples.

**Figure 6:**
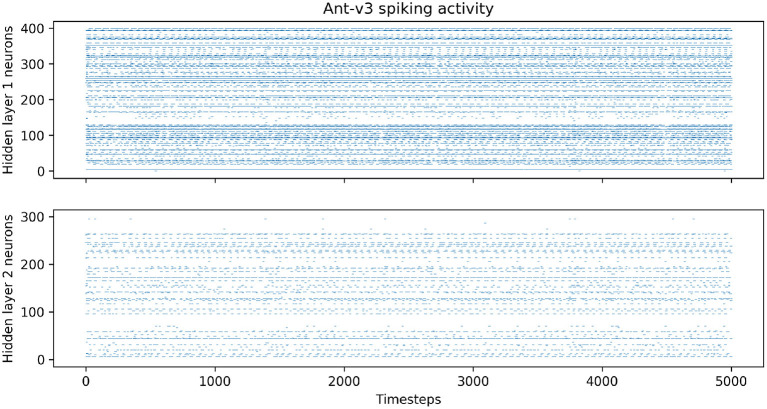
Spiking activity in the both hidden layers during one Ant-v3 episode. The x-axis shows the total number of time steps (1,000 environment time steps multiplied by five SNN time steps for each observation).

After training, we analyzed the trained networks' spiking activities during evaluation, and measured the average firing rates of the neurons in the hidden layers over 100 episodes. Figure 6 shows the spiking activity during one Ant-v3 episode. The top panel, showing the activity of the 400 neurons within the first hidden layer over 5,000 time steps, has an average firing rate of 6.77%. Meaning that only 6.77% out of the total 2 million time steps (400 neurons simulated for 5,000 time steps) include a spike. The activity gets even more sparse in the second hidden layer, shown on the bottom panel of Figure 6. There, the average firing rate measured across all 300 neurons is 1.69%. The average firing rates measured on all environments are summarized in Table 4.

**Table 4:** Average firing rates in hidden layers one and two, measured for each environment over 100 evaluation episodes.

**Environment**	**Average firing rate**	

	**Hidden layer 1**	**Hidden layer 2**
Ant-v3	6.77%	1.69%
HalfCheetah-v3	10.99%	6.87%
Hopper-v3	5.77%	7.88%
Pendulum-v0	3.12%	12.86%

## 5. Discussion and conclusion

In this paper we demonstrated that SNNs can be used as function approximators in DRL algorithms for discrete and continuous action space problems. Our results indicate that our previous encoding and decoding methods used to solve discrete-action control problems, can be considered for continuous control problems as well. Other methods in SNN training for continuous control rely on population encoding and decoding (Tang et al., 2021). Population coding increases the number of input and output neurons based on the input and output population sizes, respectively. This increase, especially for a large population size, leads to an increased number of parameters within the network. It is also directly proportional to the number of observations, the number of actions, and the number of neurons in the first and last hidden layers. For example, using a population coded network to train the Hopper-v3 environment with a population size of 10 leads to a 38% increase in the number of trainable parameters in the network. A more extreme example is the Ant-v3 environment, because of the increased number of observations (11 for Hopper-v3 vs. 111 for Ant-v3). There, a population size of 10 leads to a 252.51% increase in the number of trainable parameters. Our method does not rely on population coding and thereby requires less trainable parameters. This is especially advantageous when deploying such networks on neuromorphic chips, where the capacity of neurons and synapses per chip is limited.

Moreover, we demonstrated that randomizing the membrane parameters across the entire network leads to faster training and to higher average rewards. It is well established that one major difference between biological neural networks and deep neural networks is the lack of cell type diversity in the latter. With the randomization effects shown here, we introduce a slight diversity in the neuron types. Looking forward, this randomization can be extended to include not only different membrane parameters for each neuron, but different neuron models in one network.

As the results of the membrane parameter search indicate, training results are highly sensitive to the choice of the membrane parameters. Even though we identify best practices to reduce the sensitivity to the hyperparameters, e.g., through increasing the simulation time and applying a two-neuron input encoding, further studies are required to identify other potential best practices when choosing membrane parameters for particular problems. For example, similar studies investigating using different values for the current and voltage decay factors, or incorporating refractory periods and reset potential values, may yield additional insights. Moreover, the insights drawn from the hyperparameter study conducted here are limited to our chosen encoding and decoding methods. Running the same grid search with different encoding and decoding techniques will yield different results, and may require different parameter ranges. For example, if a rate-coding method was chosen, whether for encoding or decoding, small simulation times as the ones considered here (e.g., three or four), will not be sufficient to capture intricate differences in continuous observations. In the future, we would like to explore whether a temporal coding mechanism, e.g., spike-latency coding or inter-spike interval coding, would be suitable for continuous control problems. One advantage of such approaches would be more sparse firing, and therefore increased energy efficiency. Temporal coding schemes, however, might require longer simulation times than the ones considered here, in order to fully represent the entire input space in continuous control problems. Furthermore, we only considered stateless neurons in this work, meaning that after every inference step, we reset the entire network state before feeding in new inputs. Another potential direction for future research is to consider using stateful neurons instead. Using stateful neurons would eliminate the need for a reset operation after each inference step, providing a potential advantage.

Our grid search on the membrane parameters focused only on the maximum reward achieved during training, i.e., on whether the network can solve the problem with the given membrane parameters. While choosing this as a metric to evaluate membrane parameter combinations is important, other metrics can provide more insights about the quality of the training and should be considered for future studies. In particular, the speed with which the network reaches the best reward (i.e., the learning speed), or the number of spikes generated can be considered as additional metrics to evaluate membrane parameter combinations. If two parameter combinations can solve the problem within the same number of episodes, then the parameter combination producing less spikes should be preferred.

While DRL algorithms have been successful in solving complex tasks, whether in game playing or in robotics, they still suffer from generalization, reproducibility (Henderson et al., 2018), and sim2real transfer in robotics. With the constantly improving performance of SNNs on information processing tasks, the increased energy efficiency, the additional biological learning rules that can be utilized alongside backpropagation (Akl et al., 2022), and the recent integration of event-based sensors for RL tasks (Rizzo et al., 2022), we believe that SNNs can potentially tackle some of the limitations of DRL.

## Data availability statement

The code used for experiments conducted in this study is available at: https://github.com/mahmoudakl/dsrl.

## Author contributions

MA, DE, FW, and AK brought up the core concept of the paper. MA and DE conducted the experiments and analyzed the results. MA, DE, and FW wrote the paper. All authors contributed to the article and approved the submitted version.

## Footnotes

^1^ https://www.gymlibrary.dev/environments/mujoco/
